# Effect of rodent density on tick and tick-borne pathogen populations: consequences for infectious disease risk

**DOI:** 10.1186/s13071-020-3902-0

**Published:** 2020-01-20

**Authors:** Aleksandra I. Krawczyk, Gilian L. A. van Duijvendijk, Arno Swart, Dieter Heylen, Ryanne I. Jaarsma, Frans H. H. Jacobs, Manoj Fonville, Hein Sprong, Willem Takken

**Affiliations:** 1 Laboratory of Entomology, Wageningen University and Research Centre, Wageningen, The Netherlands; 2 Centre for Infectious Disease Control, National Institute for Public Health and the Environment, Antonie van Leeuwenhoeklaan 9, 3721 MA Bilthoven, The Netherlands; 3 Interuniversity Institute for Biostatistics and statistical Bioinformatics, Hasselt University, Diepenbeek, Belgium; 4 Department of Ecology and Evolutionary Biology, Princeton University, 106A Guyot Ln, Princeton, NJ 08544 USA

**Keywords:** Disease risk, *Ixodes ricinus*, Tick-borne pathogens, Transmission dynamics, Rodent density

## Abstract

**Background:**

Rodents are considered to contribute strongly to the risk of tick-borne diseases by feeding *Ixodes ricinus* larvae and by acting as amplifying hosts for pathogens. Here, we tested to what extent these two processes depend on rodent density, and for which pathogen species rodents synergistically contribute to the local disease risk, i.e. the density of infected nymphs (DIN).

**Methods:**

In a natural woodland, we manipulated rodent densities in plots of 2500 m^2^ by either supplementing a critical food source (acorns) or by removing rodents during two years. Untreated plots were used as controls. Collected nymphs and rodent ear biopsies were tested for the presence of seven tick-borne microorganisms. Linear models were used to capture associations between rodents, nymphs, and pathogens.

**Results:**

Investigation of data from all plots, irrespective of the treatment, revealed a strong positive association between rodent density and nymphal density, nymphal infection prevalence (NIP) with *Borrelia afzelii* and *Neoehrlichia mikurensis*, and hence DIN’s of these pathogens in the following year. The NIP, but not the DIN, of the bird-associated *Borrelia garinii*, decreased with increasing rodent density. The NIPs of *Borrelia miyamotoi* and *Rickettsia helvetica* were independent of rodent density, and increasing rodent density moderately increased the DINs. In addition, NIPs of *Babesia microti* and *Spiroplasma ixodetis* decreased with increasing rodent density, which had a non-linear association with DINs of these microorganisms.

**Conclusions:**

A positive density dependence for all rodent- and tick-associated tick-borne pathogens was found, despite the observation that some of them decreased in prevalence. The effects on the DINs were variable among microorganisms, more than likely due to contrasts in their biology (including transmission modes, host specificity and transmission efficiency). The strongest associations were found in rodent-associated pathogens that most heavily rely on horizontal transmission. Our results draw attention to the importance of considering transmission mode of a pathogen while developing preventative measures to successfully reduce the burden of disease.
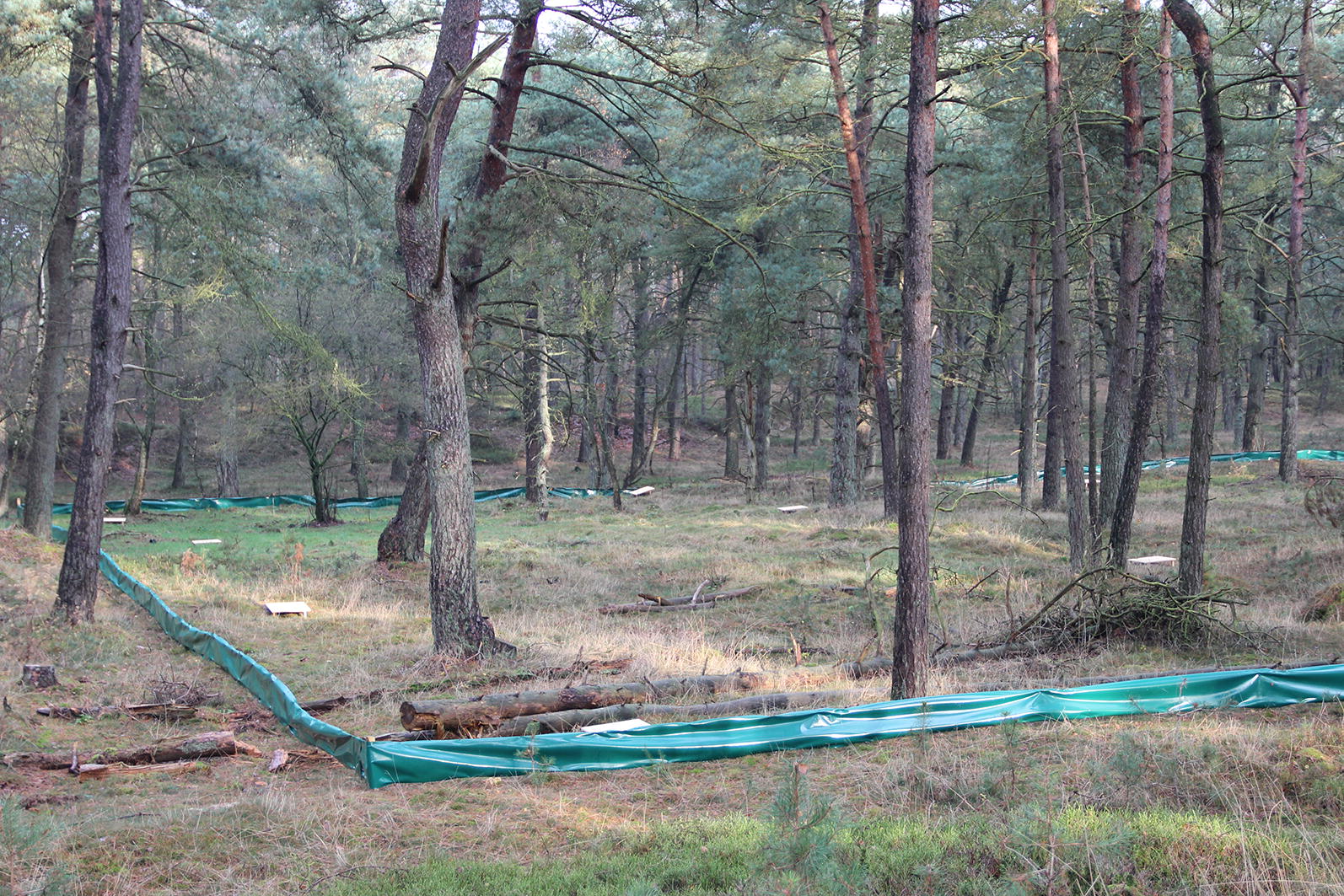

## Background

Lyme borreliosis is the most prevalent tick-borne disease in the northern hemisphere with increasing incidence and expanding endemic regions [1, 2]. The risk of acquiring Lyme borreliosis is partially determined by the density of questing ticks infected with its causative agent, *Borrelia burgdorferi* (*sensu lato*) [3, 4]. Particularly, the density of infected nymphs (DIN) is of interest, because humans are predominantly exposed to and infected with Lyme spirochetes, as well as other pathogens, by nymphs [5]. The density of infected questing ticks is a product of the density of questing ticks and infection prevalence of a pathogen, which both express high temporal variations, presumably attributed to changes in weather conditions and fluctuations in the abundance of vertebrate hosts [6–8]. The mechanisms underlying these variations are complex, as climatic conditions, vertebrate hosts and their food source, ticks, and tick-borne microorganisms form biological networks with multiple direct and indirect interactions [9]. Therefore, quantifying these interactions will help us to understand changes in the distribution and incidence of Lyme borreliosis and other tick-borne diseases.

The most common vectors of tick-borne diseases in the northern hemisphere are ticks of the *Ixodes ricinus* complex. Their survival primarily depends on their ability to find a vertebrate host, which may vary between life stages. In forested areas, larvae of the *I. ricinus* complex feed predominantly on rodents, nymphs on rodents and birds, and adults on ungulates, mostly deer [10, 11]. Although presence of deer is generally responsible for high abundance of ticks [12], variations in the density of nymphs (DON) has been associated with the density of rodents [13]. For instance, the density of host-seeking *I. scapularis* nymphs was correlated with abundance of white-footed mice in the previous year. White-footed mice are the main hosts for larval *I. scapularis*; high abundance of these mice provides more opportunities for larvae to feed successfully, and subsequently emerge as nymphs in the following year.

The abundance of rodent species is affected by many different factors, such as predation, vegetation cover, and food availability [10, 14, 15]. A key food supply for rodents is acorns and its seasonal availability has been shown to be responsible for the fluctuations in rodent densities between years and geographical locations [16–18]. In general, acorn availability increases the length of the breeding season and facilitates winter survival of forest rodents resulting in a higher rodent density in the following spring [9, 19–22]. As a consequence, in the temperate zone, an increased rodent density has been shown to cause upsurges in Puumala hantavirus disease in humans [23, 24]. In addition, several North American studies have suggested that acorns and rodents are good predictors for Lyme-disease risk because rodents are reservoir hosts of *B. burgdorferi* (*s.l.*) [9, 15, 25]. The causal relationship between rodent fluctuations and Lyme disease incidence, however, remains unresolved as this has not been investigated in experimental settings, enabling the exclusion of confounding factors.

In the Netherlands, the wood mice (*Apodemus sylvaticus* Linnaeus) and bank voles (*Myodes glareolus* (Schreber)) are amplifying hosts of several tick-borne pathogens including *B. afzelii*, *B. miyamotoi*, *Babesia microti* and *Neoehrlichia mikurensis* [26–30], and the most common hosts of larval *I. ricinus* [31]. Apart from rodent-borne pathogens mentioned above, *I. ricinus* carries many other microorganisms including *B. garinii*, *Spiroplasma ixodetis* and *Rickettsia helvetica* [32]. Most, if not all, of the pathogens are transmitted between ticks *via* a vertebrate host (horizontally), which can be broadly divided into co-feeding and systemic transmission (Table 1). Co-feeding relies on localized and temporal infection in the vertebrate skin and occurs when infected and uninfected ticks feed close to each other [33, 34]. Systemic transmission depends more on a persistent infection in a host, which can be local (e.g. skin) or systemic (e.g. blood) [34]. Amplifying hosts are responsible for producing infected ticks and therefore, for increasing risk of human exposure. In addition, ticks maintain microorganisms such as *S. ixodetis via* vertical transmission, with different efficiency (Table 1). Some bacteria such as *R. helvetica* and *B. miyamotoi*, can utilize both horizontal and vertical transmission routes [35, 36]. It is unclear how variations in rodent densities affect the disease risk of tick-borne pathogens with different transmission modes, particularly in the European setting.

**Table 1:** Transmission modes and amplification hosts of tick-borne microorganisms

Microorganism	Transmission mode	Proposed amplification host
*Babesia microti*	Horizontal [91]	Rodents [93]
*Borrelia afzelii*	Horizontal [62]	Rodents [55]
*Borrelia garinii*	Horizontal [62]	Birds [94]
*Borrelia miyamotoi*	Horizontal/vertical [74]	Rodents [74]
*Neoehrlichia mikurensis*	Horizontal [51]	Rodents [51]
*Rickettsia helvetica*	Horizontal/vertical [44]	Birds [36]
*Spiroplasma ixodetis*	Vertical/horizontal [92; this study]	Rodents [this study]

The goal of the present study was to investigate how rodent densities, the density of *I. ricinus* nymphs and transmission dynamics of tick-borne pathogens interact in order to generate the density of infected ticks. To our knowledge, this is the first European study experimentally investigating these relationships in the field. In addition, no prior study has assessed the influence of rodent density on prevalence and density of tick-borne microorganisms other than rodent-borne. Our approach was to artificially manipulate the rodent densities by either acorn addition or rodent removal for two consecutive years in a natural habitat. We measured and quantified the rodent, nymph, and pathogen population responses to these treatments, as well as performed regression analysis. Using this approach, we aimed to learn whether rodent densities play a major role in shaping the density of questing ticks and transmission dynamics of tick-borne microorganisms, which in turn, will help assess and potentially predict disease risk and formulate possible intervention strategies.

Given that rodents are locally the most substantial hosts for larvae [31] and high rodent density results in high larval encounter rates, increase of the rodent density at a given year_*t*_ is expected to lead to a rise in density of nymphs in the following year (DON_*t+*1_). Along with the higher rodent densities, transmission events of tick-borne microorganisms are expected to increase. We anticipate that differences in the microorganisms’ modes of transmission as well as host amplification potential are main determinants in the change after manipulation. Our hypothesis is that the NIP_*t+*1_ (nymphal infection prevalence) of tick-borne pathogens, such as *B. afzelii*, *N. mikurensis* and *B. microti*, which are amplified by rodents, is dependent on the density of rodents. Consequently, we expect a synergistic effect of rodent densities on the density of infected nymphs one year later (DIN_*t+*1_). Also, we hypothesise that rodent densities will not alter the NIP_*t+*1_ of tick-associated microorganisms, such as *R. helvetica*, *B. miyamotoi* and *S. ixodetis*, which predominantly rely on vertical transmission. Further, we expect that DIN_*t+*1 *R. helvetica*_, DIN_*t+*1 *B. miyamotoi*_ and DIN_*t+*1 *S. ixodetis*_ will be only moderately affected by increasing rodent density. In case of *B. garinii*, a tick-borne pathogen amplified by birds [37, 38], we expect that increasing rodent density will increase the proportion of larvae feeding on rodents and, therefore, have a negative effect on NIP_*t+*1 *B. garinii*_. Lastly, we hypothesize that a higher rodent density will have no effect on DIN_*t+*1 *B. garinii*_.

## Methods

### Study sites

The study was conducted at the forest reserves Planken Wambuis (52°01′45″N, 5°48′49″E) and Noord Ginkel (52°02′23″N, 5°45′09″E) near Wageningen, The Netherlands. Both forests are dominated by Scots pine (*Pinus sylvestris*) and harbour a diversity of bird and mammal species, including wood mice (*A. sylvaticus*), bank voles (*M. glareolus*), common shrews (*Sorex araneus*), wild boar (*Sus scrofa*), roe deer (*Capreolus capreolus*), red deer (*Cervus elaphus*) and a few free-ranging cattle and horses.

### Manipulation of rodent density and estimation of nymphal density

In both forests, six plots of 50 × 50 m were selected with at least 350 m between plots (Additional file 1: Figure S1). Each plot was assigned to one of three treatments (rodent removal, control or acorn addition). In rodent removal plots, rodents were trapped for one night a month with Heslinga live traps (Heslinga Traps, Groningen, The Netherlands) in a 5 × 5 grid with 10 m inter-trap distance. Captured rodents were euthanized by cervical dislocation. The first rodent removal event was directly after the mark-recapture trapping in September 2012. Thereafter, rodents that accidentally found their way into the plots were removed monthly until December 2014 using the same grid with traps. Four control plots received no treatment. To increase rodent density, acorns were added to four plots [39]. Acorns were provided beneath feeding stations, which were made of 60 × 60 cm plates kept 5 cm above the ground to prevent acorn predation by birds and large mammals. In each plot, 16 of these feeding stations were placed in a 4 × 4 grid with 15 m between feeding stations. Each feeding station was provided with 6.25 kg of acorns in November and January of 2012 and 2013 (1600 kg in total). Control feeding stations without acorns were also placed in the control and rodent removal plots. A plastic screen, 40 cm high and dug 10 cm into the ground was placed as a barrier around the four rodent removal plots to prevent immigration of rodents (Additional file 1: Figure S1). To overcome a possible bias in large vertebrate community caused by a visual effect, screens were also placed around the control and acorn addition plots. However, the lowest 10 cm of these screens was left open to enable rodents to walk in and out freely.

Tick density was estimated monthly in each plot by blanket dragging over the vegetation. At each plot, a 1 m^2^ blanket was dragged over four transits of 50 m and inspected at 25 m intervals. All attached nymphs were counted. Dragging was performed in the afternoons (12:00–18:00 h CET) when the vegetation was dry. Given that nymphs have been shown to quest when the weekly mean daily maximum temperature exceeds 7 °C [40–42], we included temperature data from September 2012 to December 2015 to investigate the relationship between temperature and onset of tick activity. Daily measurements were collected from the nearest weather station (Deelen, KNMI, the Netherlands; Additional file 4: Table S1).

### Rodent samples and nymph collection

Rodents were sampled at three-month intervals (March, June, September and December) from September 2012 until December 2014. At each plot, 25 Heslinga live traps were placed in a 5 × 5 grid. Traps were pre-baited with oats for 3 days, after which they were rebaited with grain, carrot and mealworms and set at 9:00 h CET. Traps were then inspected four times at 12-h intervals. Trapped rodents were marked by shaving a patch of fur from their side [43]. Rodent density was calculated per species according to the Schnabel method (multiple marking; [44, 45]. During the morning trappings, newly captured rodents were screened for ticks, and larvae were counted. A small ear biopsy was taken with sterile scissors from each newly captured rodent and stored in 70% ethanol at − 20 °C until further analysis.

Questing nymphs were collected during monthly density estimation. All nymphs attached to the blanket were collected and stored individually in 70% ethanol at − 20 °C until further analysis.

### DNA extraction and pathogen detection

Ear biopsies and nymphs were analysed individually. DNA from a maximum of 40 nymphs per plot per month was extracted with ammonium hydroxide as described previously [46]. DNA from the ear biopsies was extracted using the Qiagen DNeasy Blood & Tissue Kit according to the manufacturer’s protocol (Qiagen, Venlo, The Netherlands). The lysates were stored at 4 °C. Samples were analysed with different (multiplex) real-time PCRs, based on various target genes depending on microorganism of interest such *B. burgdorferi* (*s.l*.) [47], *B. miyamotoi* [48], *N. mikurensis* [49], *R. helvetica* [50], *B. microti* and *S. ixodetis* (this study, Additional file 4: Text S1). A detailed description of the qPCR protocol is provided in Additional file 4: Text S1. Samples positive for *B. burgdorferi* (*s.l*.), were subjected to conventional PCR followed by sequencing to identify a genotype [47].

### Data analysis and modelling

Data analysis and model building were performed in R version 3.5.1 [51] and RStudio [52]. To evaluate whether rodent removal and acorn addition treatments were successful, we compared means of densities of rodents (data from 2013 and 2014) between the treatments using the non-parametric Wilcoxon signed-rank test. The same test was used to evaluate whether the treatments influenced the DON, and density of rodent-associated pathogens, *B. afzelii* and *N. mikurensis* (data from 2014 and 2015). Since monthly at each plot 200 m^2^ were inspected for questing nymphs, we combined these measurements into a yearly DON per 2400 m^2^ (by summing up all nymphs from 12 months). The differences in prevalence of microorganisms and tick burdens between two rodent species were compared with the Chi-square test and non-parametric Wilcoxon signed-rank test, respectively.

To investigate how well the density of rodents from 2013 and 2014 predicts DON_*t+*1_, NIP_*t+*1_ and DIN_*t+*1_, we performed regression analyses. Several linear models for DON_*t+*1_ (the annual median) were assessed with different interactions between rodent density, year, and treatment. For NIP_*t+*1_, binomial generalized linear models were assessed with different interactions between rodent density and year. Because NIP is represented by fraction data, we choose a binomial generalized linear model taking into account sample size with the logit link transform. For DIN_*t+*1_, linear models were assessed with different interactions between rodent density and year. DIN data were calculated by multiplying DON and NIP, which are both potentially influenced by rodent density and therefore we have also included (rodent density)^2^ as a covariate.

Year (2013, 2014) and treatment (acorn addition, control and rodent removal) were categorical variables while DON, NIP and DIN were numerical variables. The ranges of DON, NIP and rodent density are provided in Additional file 4: Table S2 and Table S3, respectively. For all models, best-fitting models were compared on the basis of a likelihood ratio test, *R*^2^ (linear models) and AIC (linear and generalized models). Model selection was performed using histograms to visually evaluate normality of the residuals. If there clearly was no best model, the simpler model was selected.

## Results

### Effect of treatment on rodent density, DON, DIN_*B. afzelii*_, and DIN_*N. mikurensis*_

**Fig. 1:**
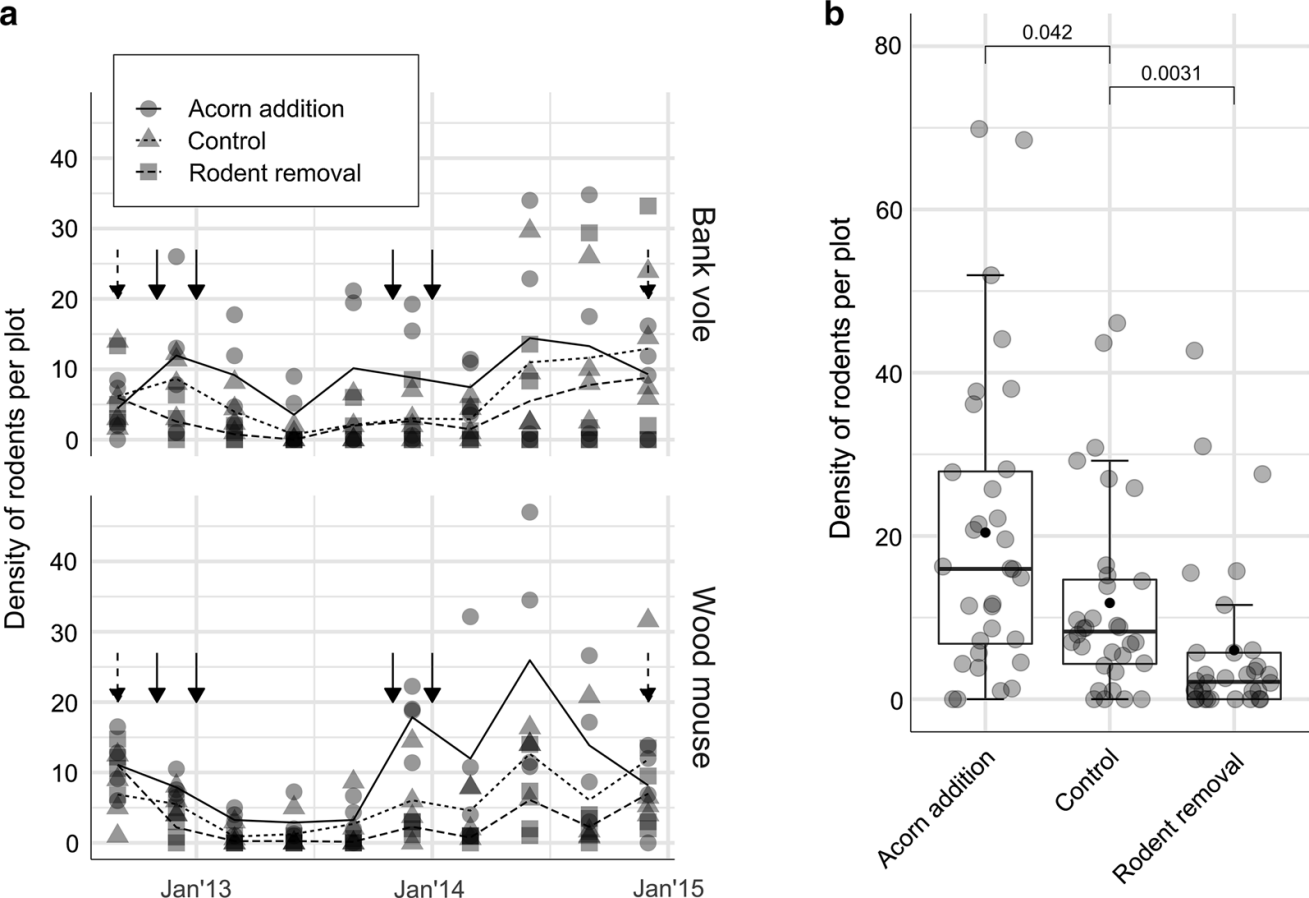
**a** Mean density of two rodent species, bank vole and wood mouse per plot. Solid arrows indicate events of acorn supplementation (November and January); dashed arrows indicate when monthly removal of rodents started (September 2012) and ended (December 2014). **b** Box plots of rodent density per plot for each treatment (data from 2013 and 2014). The lower and upper hinges correspond to the first and third quartiles (the 25th and 75th percentiles). The upper whisker shows the largest value no further than 1.5 * IQR from the hinge (where IQR is the inter-quartile range, or distance between the first and third quartiles) and the lower whisker shows the smallest value at most 1.5 * IQR of the hinge. The differences in the rodent density between the treatments was calculated based on the mean (black dot) with the Wilcoxon test and the overall difference is statistically significant (*P* < 0.0001). The diagram shows also the median observation (solid horizontal line)

Rodent density was affected by treatment (Fig. 1). With bank voles the effects were apparent throughout the intervention period, while with wood mice addition of acorns led to a strong increase in density in the second year of the study. The removal of rodents led to a lower (*P* = 0.0031) rodent density and the addition of acorns led to a higher (*P* = 0.042) rodent density than in the control plots in years 2013 and 2014 (Fig. 1).

**Fig. 2:**
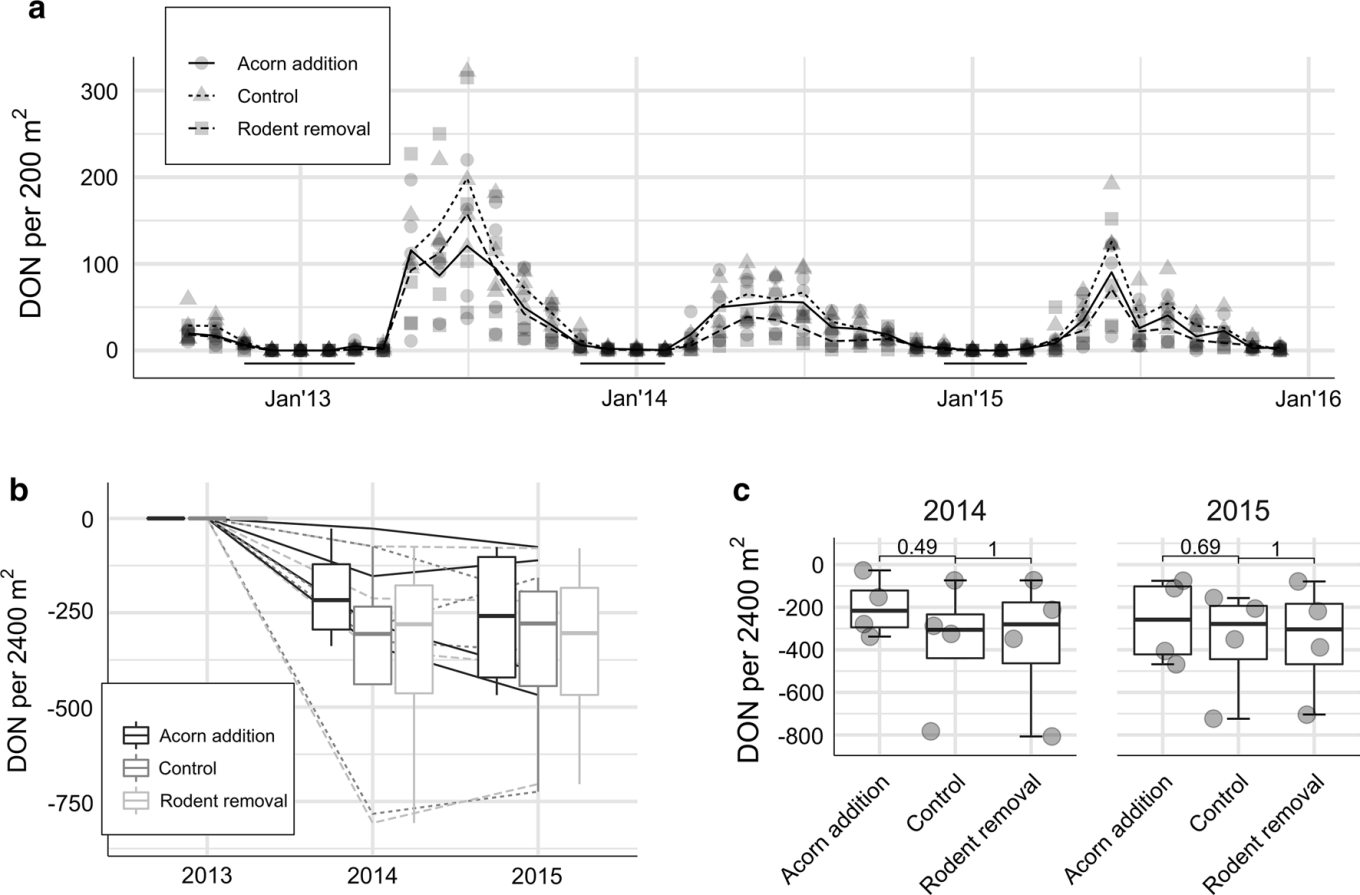
**a** Mean density of questing nymphs (DON) per 200 m^2^. Horizontal solid lines just above the x-axis depict months with average temperature below 7 °C. In winter 2012/2013, the number of months with the mean temperature below 7 °C was five, while in both 2013/2014 and 2014/2015 was four, however different months. **b** Density of nymphs (DON) in 2014 and 2015 in all three treatments in comparison to 2013 (baseline year). **c** Differences in DON between the treatments in two separate years calculated with the Wilcoxon test with a correction for a baseline year (2013). The overall differences between the treatments were not significant either in 2014, or 2015 (*P* > 0.59 and *P* > 0.87, respectively)

The DON fluctuated over the years and was the highest from May until October (Fig. 2). We observed that the moment that nymphs started to quest was in the first month of the year with a mean temperature above 7 °C. The number of months with a mean temperature below 7 °C varied between the years (Fig. 2, Additional file 2: Figure S2). In 2013, five months had mean temperatures below 7 °C, whereas both 2014 and 2015 had three months with mean temperatures below 7 °C, but these were spread differently throughout the year. The mean DON of all plots in 2013, 2014 and 2015 were 581, 272 and 257 per 2400 m^2^ (200 m^2^ × 12 months), respectively. Mean nymphal density in 2014 and 2015 was significantly lower than in 2013 (*P* = 0.0083 and *P* = 0.013, respectively), whereas the mean nymphal densities of 2014 and 2015 were not significantly different (*P* = 0.63; not shown). There was no effect (*P* = 0.27) of acorn addition and a negative effect (*P* = 0.043) of rodent removal on the DON in the same (not shown) or following years. Nevertheless, when the density of nymphs from 2013 served as a baseline to measure the effect of a treatment on the DON in 2014 and 2015, there was no significant effect (Fig. 2).

**Fig. 3:**
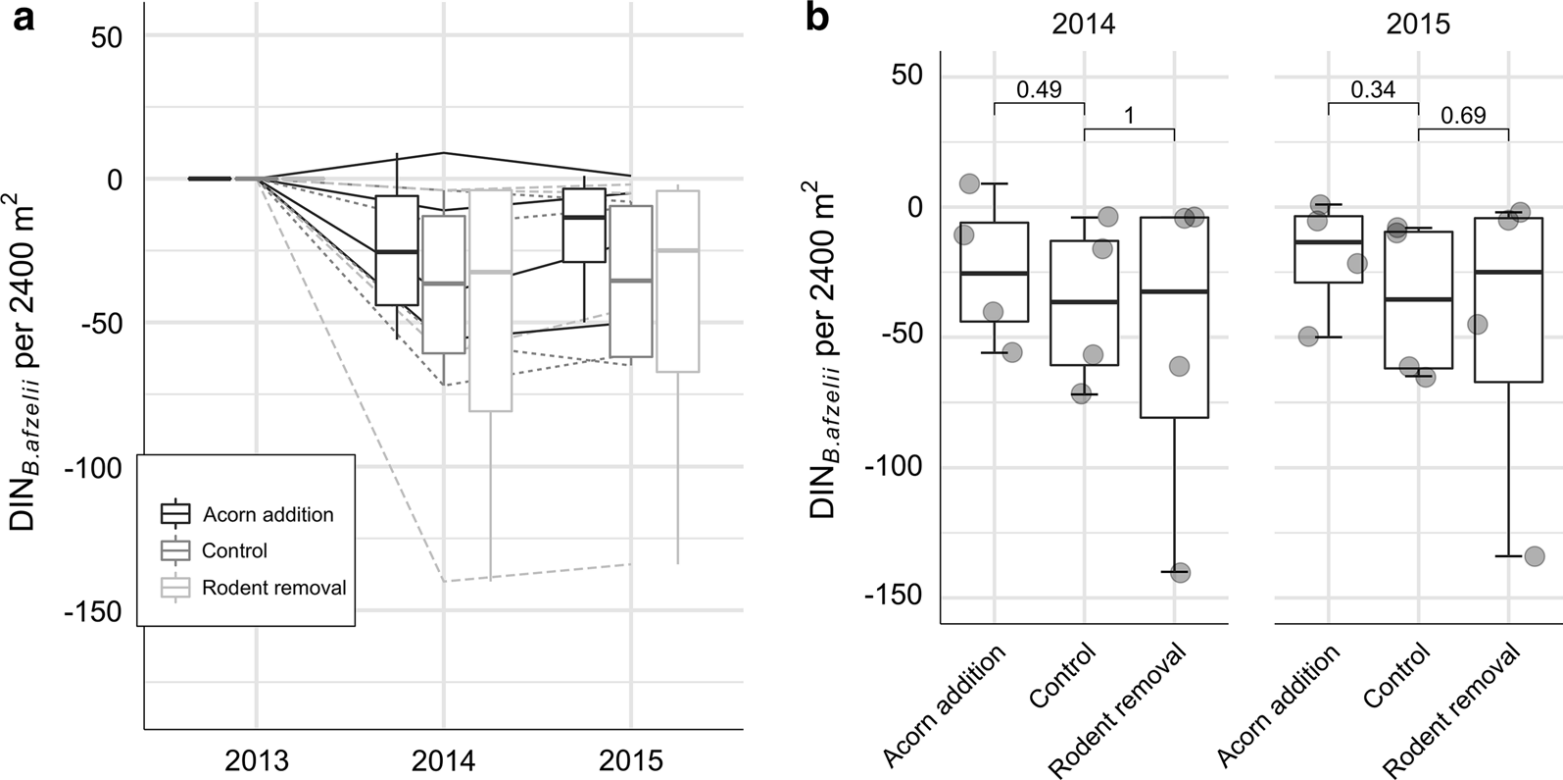
**a** Density of nymphs infected with *B. afzelii* (DIN _*B. afzelii*_) in 2014 and 2015 in all three treatments in comparison to 2013 (baseline year). **b** Differences in DIN _*B. afzelii*_ between the treatments in two separate years calculated with the Wilcoxon test with a correction for a baseline year (2013). The overall differences between the treatments were not significant either in 2014, or 2015 (*P* = 0.69 and *P* = 0.53, respectively)

To investigate the effect of treatment on the dynamics of tick-borne pathogens amplified by rodents, we compared the mean DIN_*B. afzelii*_ and DIN_*N. mikurensis*_ between the treatments in 2014 and 2015. Our analyses before and after a correction for a baseline DIN from 2013 showed that there was no effect of either acorn addition or rodent removal on the density of nymphs infected with *B. afzelii* and *N. mikurensis* in the following years (Fig. 3 and Additional file 3: Figure S3).

### Rodent sample and nymph collection

A total of 2386 rodents was caught in the experiment. From these, 345 bank voles and 547 wood mice were inspected for ticks, from which 155 and 346 were infested with larvae, respectively. The average number of larvae found on wood mice (9.0; 95% CI: 7.6–10.4) was significantly higher (*W* = 118,520, *P* < 0.0001) than the average in bank voles (4.2; 95% CI: 3.0–5.4). None of the bank voles and 97 wood mice were infested with nymphs and the average nymphal burden was 0.2 (95% CI: − 0.2–0.6).

A total of 772 ear biopsies was taken (478 from wood mice and 294 from bank voles) and subjected to pathogen detection. In addition, 13,916 nymphs were collected by dragging, from which 7609 were tested for the presence of tick-borne pathogens. A detailed overview of rodent densities, number of analysed rodents, tick density and analysed ticks per treatment, month, and year are provided in Additional file 4: Table S2 and Table S3.

### Pathogen detection

**Fig. 4:**
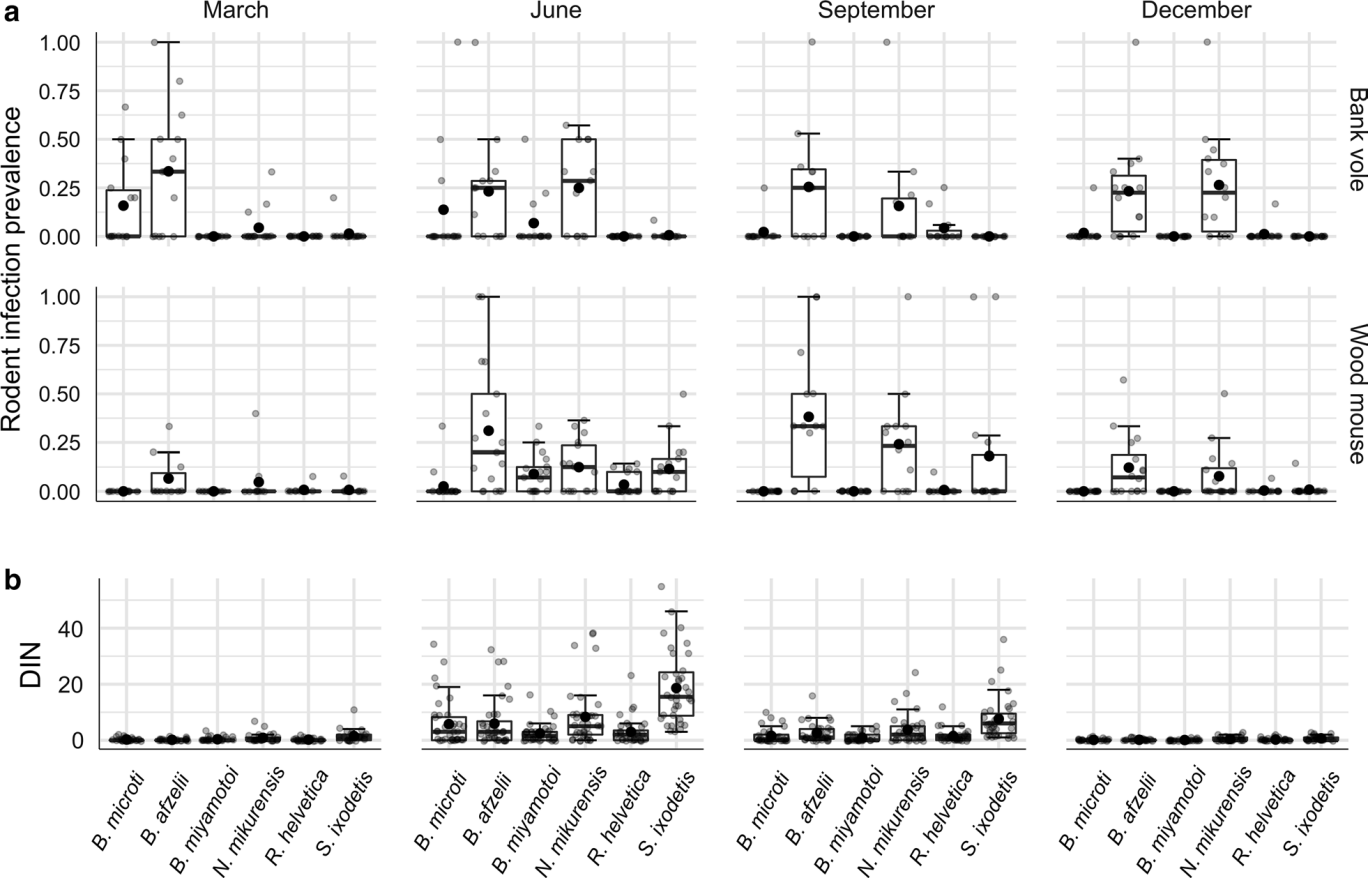
Overview of tick-borne microorganism infections in rodents and nymphs. **a** Rodent infection prevalence separately for each collection month and rodent species. **b** Density of infected nymphs (DIN) separately for each collection month (data combined from 2013 and 2014)

In the rodent ear biopsies and the collected questing nymphs we detected DNA of *B. burgdorferi* (*s.l.*), *B. miyamotoi*, *N. mikurensis*, *B. microti*, *R. helvetica* and *S. ixodetis* (Fig. 4). The sequencing success of qPCR-positive ticks (*n* = 1017) for *B. burgdorferi* (*s.l.*) was 64%, and four genospecies were identified: *B. afzelii*, *B. garinii*, *B. valaisiana* and *B. burgdorferi* (*s.s.*). *Borrelia*-positive rodent biopsies were not sequenced and were treated as *B. afzelii* in further analysis. A justification for this assumption derives from previous studies, which have shown that, in the Netherlands, more than 99% of the positive rodents infected with *B. burgdorferi* (*s.l.*) carried *B. afzelii* [53, 54]. The prevalence of *B. afzelii* as well as *N. mikurensis* was higher in bank voles than in wood mice (*χ*^2^ =  3.296, *df* = 1, *P* = 0.0694 and *χ*^2^ = 4.234, *df* = 1, *P* = 0.0396, respectively). Interestingly, *S. ixodetis* was almost exclusively detected in wood mice with prevalence significantly higher than in bank voles (*χ*^2^ = 14.264, *df* = 1, *P* = 0.0002), whereas *B. microti* was almost exclusively found in bank voles with prevalence significantly higher than in wood mice (*χ*^2^ = 27.012, *df* = 1, *P* < 0.0001). The prevalence of *R. helvetica* was not significantly different between two rodent species (*χ*^2^ = 0.803, *df* = 1, *P* = 0.3703). A complete overview of infection prevalence of all pathogens in ticks and rodent biopsies is provided in Additional file 4: Table S4.

The only pathogen consistently present in both rodent species throughout the year was *B. afzelii* (Fig. 4). The infection in rodents persisted despite infected nymphs not being active in months below 7 °C. Other pathogens, such as *N. mikurensis*, *B. miyamotoi*, *R. helvetica* and *S. ixodetis*, were present in the rodent population mostly when activity of rodents and (infected) nymphs overlapped (Fig. 4).

### Rodent density *versus* DON_t+1_

Equations of all tested models investigating the association between rodent density and DON_*t+*1_, NIP_*t+*1_, and DIN_*t+*1_, their *R*^2^, AIC values, and results of a likelihood test are provided in Additional file 4: Table S5. Full equations of the best-fitting models are provided in Additional file 4: Table S6, while Table 2 shows significant interactions incorporated in the models as well as the type of effect rodent density had on all responses (DON_*t+*1_, NIP_*t+*1_ and DIN_*t+*1_). Because our treatments affected bank voles and wood mice simultaneously, rodent density data used in the models consist of rodent species added together.

**Table 2:** Best models for prediction of density of nymphs (DON), nymphal infection prevalence (NIP), and density of infected nymphs (DIN)

Eq. no.	Response	Equation	Type	Year	Trend
1	DON_*t+*1_	${ = 1} . 2 8\times {\text{rodent density }} - 8. 7 5\times I_{\text{year = 2014}} + 1 1. 7 7\times I_{\text{treatment = control}}$	LM	–	↑***
2	NIP_*t+*1_ *B. afzelii*	$= - 3.25 + 0. 0 2\times {\text{rodent density}}$	GLM, binomial	–	↑***
3	DIN_*t+*1_ *B. afzelii*	$= 5.33 + 0. 7 5\times {\text{rodent density}}$	LM	–	↑***
4	NIP_*t+*1_ *N. mikurensis*	$= - 2.85 + 0. 0 3\times {\text{rodent density}}$	GLM, binomial	–	↑***
5	DIN_*t+*1_ *N. mikurensis*	$= 6. 8 4+ 1.52 \times {\text{rodent density }}$	LM	–	↑***
6	NIP_*t+*1_ *B. miyamotoi*	$= - 3.33 + 0. 0 3\times {\text{rodent density }} \times I_{\text{year = 2014}}$	GLM, binomial	2013	↓
				2014	↑**
7	DIN_*t+*1_ *B. miyamotoi*	$= 4.88 + 0. 3 4\times {\text{rodent density}}$	LM	–	↑*
8	NIP_*t+*1_ *B. microti*	$= - 2. 9 3- 0.02 \times {\text{rodent density}}$	GLM, binomial	–	↓***
9	DIN_*t+*1_ *B. microti*	$= 2.64 - 0.04 \times \left( {\text{rodent density}} \right){}_{{}}^{2} + 1.62 \times {\text{rodent density}}$	LM	–	↑↓*
10	NIP_*t+*1_ *B. garinii*	$= - 4.28 - 0. 0 4\times {\text{rodent density }} + 0. 8 3\times I_{\text{year = 2014}}$	GLM, binomial	–	↓***
11	DIN_*t+*1_ *B. garinii*	$= 3.00 \left( {null} \right)$	LM	–	→
12	NIP_*t+*1_ *R. helvetica*	$= - 3.52 + 0.03 \times {\text{rodent density}} \times I_{\text{year = 2014}}$	GLM, binomial	2013	→
				2014	↑***
13	DIN_*t+*1_ *R. helvetica*	$= 3.21 + 0. 7 0\times {\text{rodent density}}$	LM	–	↑*
14	NIP_*t+*1_ *S. ixodetis*	$= - 1.04 - 0. 0 1\times {\text{rodent density}}$	GLM, binomial	–	↓***
15	DIN_*t+*1_ *S. ixodetis*	$= 24.94 - 0.12 \times \left( {\text{rodent density}} \right){}_{{}}^{2} + 5.88 \times {\text{rodent density}}$	LM	–	↑↓***

*Notes*: Only significant interactions are shown in the equations; full equations can be found in Additional file 4: Table S6. Arrows indicate whether an effect of rodent density was positive, negative or none. Two arrows, one going up and one going down indicate non-linear association (parabola). Asterisks denote significance of an effect (*P ≤ 0.05, **P ≤ 0.01, ***P ≤ 0.001)

**Fig. 5:**
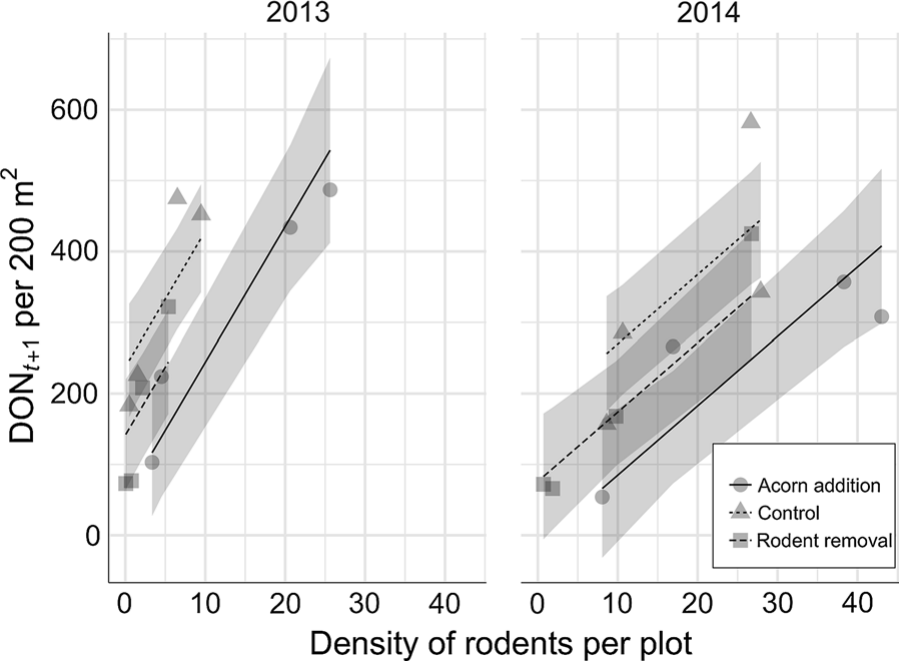
Effect of rodent density on DON_*t+*1_. The plot shows the relationships between the number of rodents per plot in year *t* and DON (number per 200 m^2^ per plot) in the following year (*t*+1). Rodent density had significant positive effect on DON in all treatments and years

The model that fit the data best indicated that rodent density and DON_*t+*1_ were significantly positively associated (*P* = 0.000631). The best model was a linear model of rodent density incorporating year and treatment as covariates explaining 61% of the variance (Table 2, Eq. 1; Fig. 5).

### Rodent density *versus* rodent-associated pathogens

**Fig. 6:**
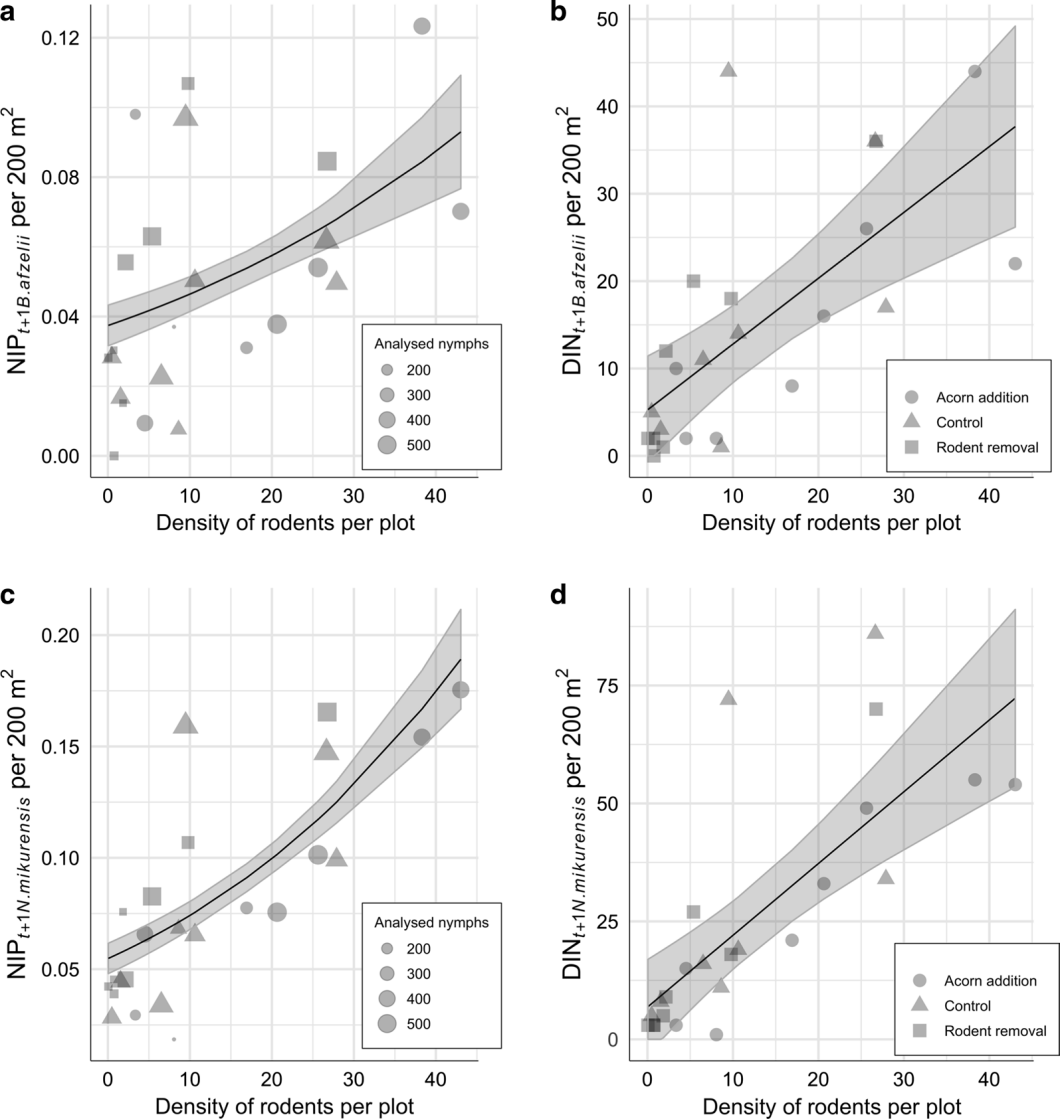
Association between density of rodents and pathogens amplified by rodents. The graphs show the relationship between the number of rodents per plot in year *t* and NIP and DIN (number per 200 m^2^ per plot) in year *t*+1. **a** Effect of rodent density on NIP_*t+*1 *B. afzelii*_. Rodent density had significant positive effect on NIP. **b** Effect of rodent density on DIN_*t+*1 *B. afzelii*_. Rodent density has significant positive effects on DIN. **c** Effect of rodent density on NIP_*t+*1 *N. mikurensis*_. Rodent density had significant positive effect on NIP. **d** Effect of rodent density on DON_*t+*1 *N. mikurensis*_. Rodent density had significant positive effect on DIN

**Fig. 7:**
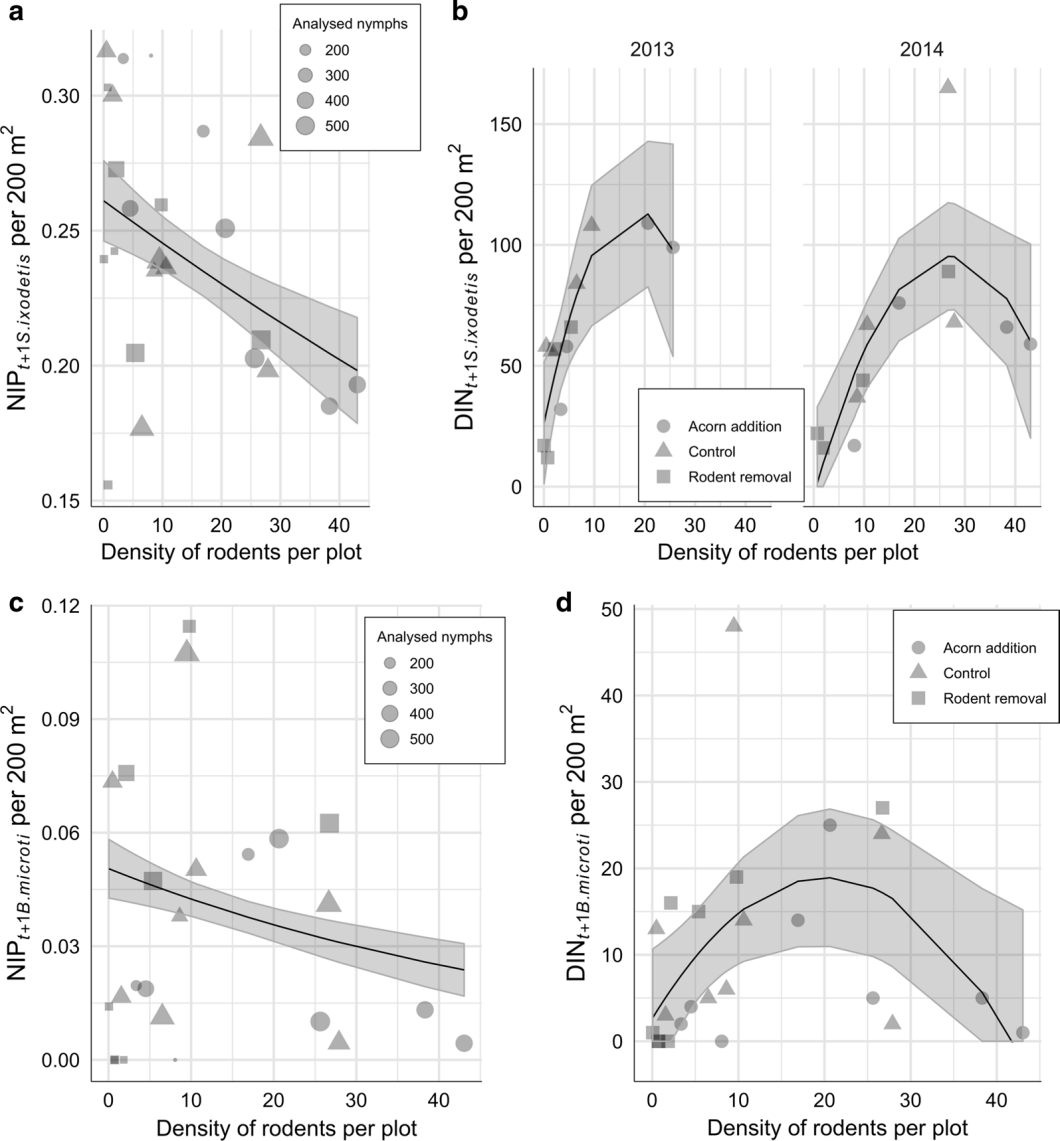
Association between density of rodents and tick-associated microorganisms. The graphs show the relationship between the number of rodents per plot in year *t* and NIP and DIN (number per 200 m^2^ per plot) in year *t*+1. **a** Effect of rodent density on NIP_*t+*1 *S. ixodetis*_. Rodent density had significant negative effect on NIP. **b** Effect of rodent density on DON_*t+*1 *S. ixodetis*_. Rodent density had significant non-linear effect on DIN. **c** Effect of rodent density on NIP_*t+*1 *B. microti*_. Rodent density had significant negative effect on NIP. **d** Effect of rodent density on DIN_*t+*1 *B. microti*_. Rodent density had significant non-linear effect on DIN

Regarding *B. afzelii* and *N. mikurensis*, there was a significant positive association between rodent density and NIP_*t+*1_ (*P* < 0.0001 and *P* < 0.0001), and rodent density and DIN_*t+*1_ (*P* = 0.000187 and *P* < 0.0001; Fig. 6). The best model for both NIP_*t+*1 *B. afzelii*_ and NIP_*t+*1 *N. mikurensis*_ was a simple generalized linear model of rodent density (Table 2, Eq. 2 and Eq. 4). In the case of DIN_*t+*1_, a simple linear model of rodent density was the best and explained 45% and 56% of the variance in DIN_*t+*1 *B. afzelii*_ and DIN_*t+*1 *N. mikurensis*_, respectively (Table 2, Eq. 3 and Eq. 5). Regarding another pathogen amplified by rodents, *B. microti*, there was a negative effect (*P* < 0.0001) of rodent density on NIP_*t+*1_ and the best model was a simple generalized linear model of rodent density (Table 2, Eq. 8; Fig. 7). In the case of DIN_*t+*1 *B. microti*_, the best model was a linear model of rodent density and (rodent density)^2^, Table 2, Eq. 9), which explained 20% of the variance. The model including a quadratic term allowed to reveal significant negative (*P* = 0.0141) non-linear association between rodent density and *B. microti* (Fig. 7).

### Rodent density *versus* a bird-associated pathogen

**Fig. 8:**
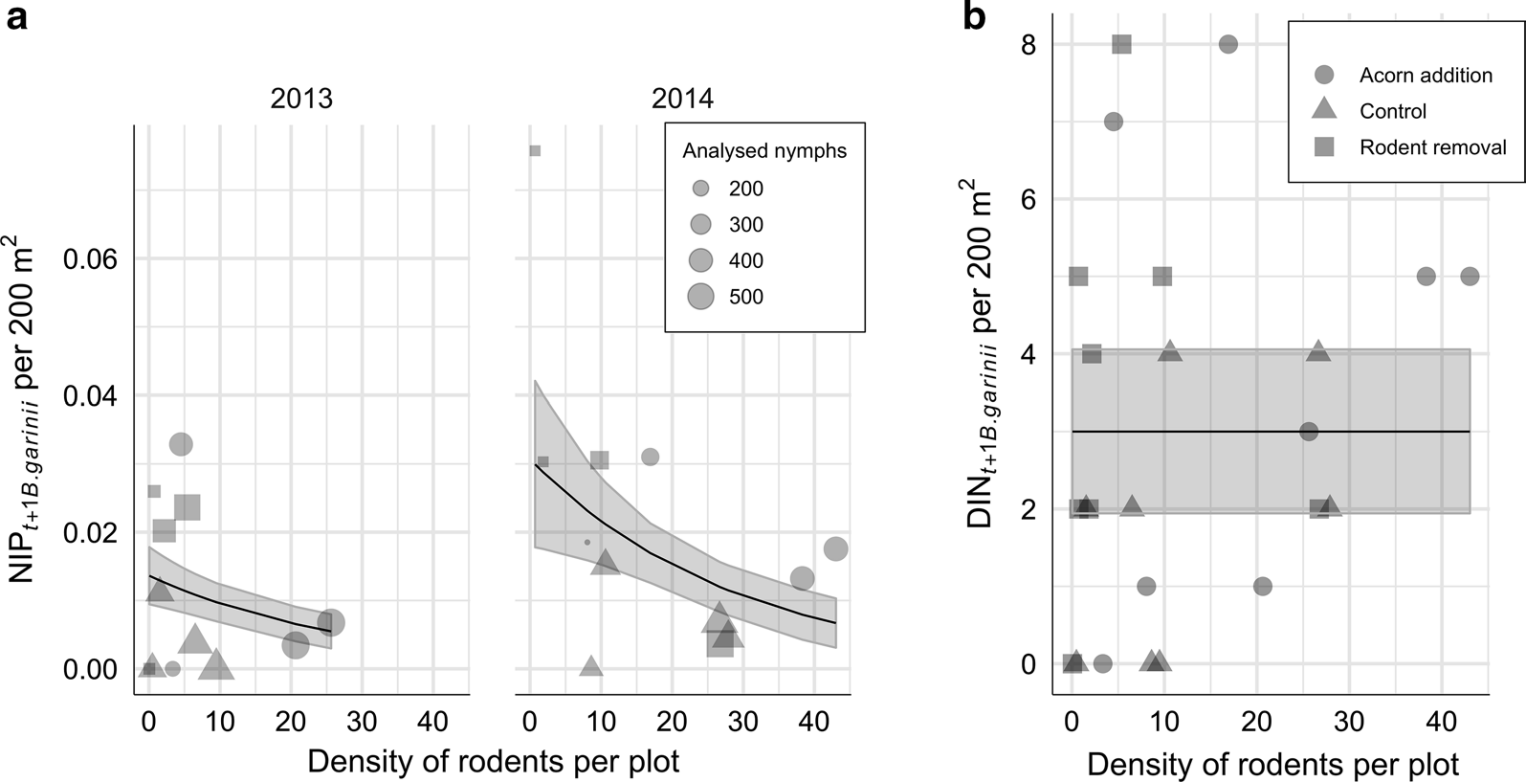
Association between density of rodents and a pathogen amplified by birds. The graphs show the relationship between the number of rodents per plot in year *t* and NIP and DIN (number per 200 m^2^ per plot) in year *t*+1. **a** Effect of rodent density on NIP_*t+*1 *B. garinii*_. Rodent density had significant negative effect on NIP in both years. **b** Effect of rodent density on DIN_*t+*1 *B. garinii*_. Rodent density had no effect on DIN

There was a significant negative association (P = 0.000149) between rodent density and NIP_*t+*1 *B. garinii*_ and no association between rodent density and DIN_*t+*1 *B. garinii*_ (Fig. 8), which remained constant through the experiment. The best model for NIP_*t+*1 *B. garinii*_ was a generalized linear model of rodent density and year (Table 2, Eq. 10), while none of the tested models for DIN_*t+*1 *B. garinii*_ was better than a null model (Table 2, Eq. 11).

### Rodent density *versus* vertically transmitted microorganisms

**Fig. 9:**
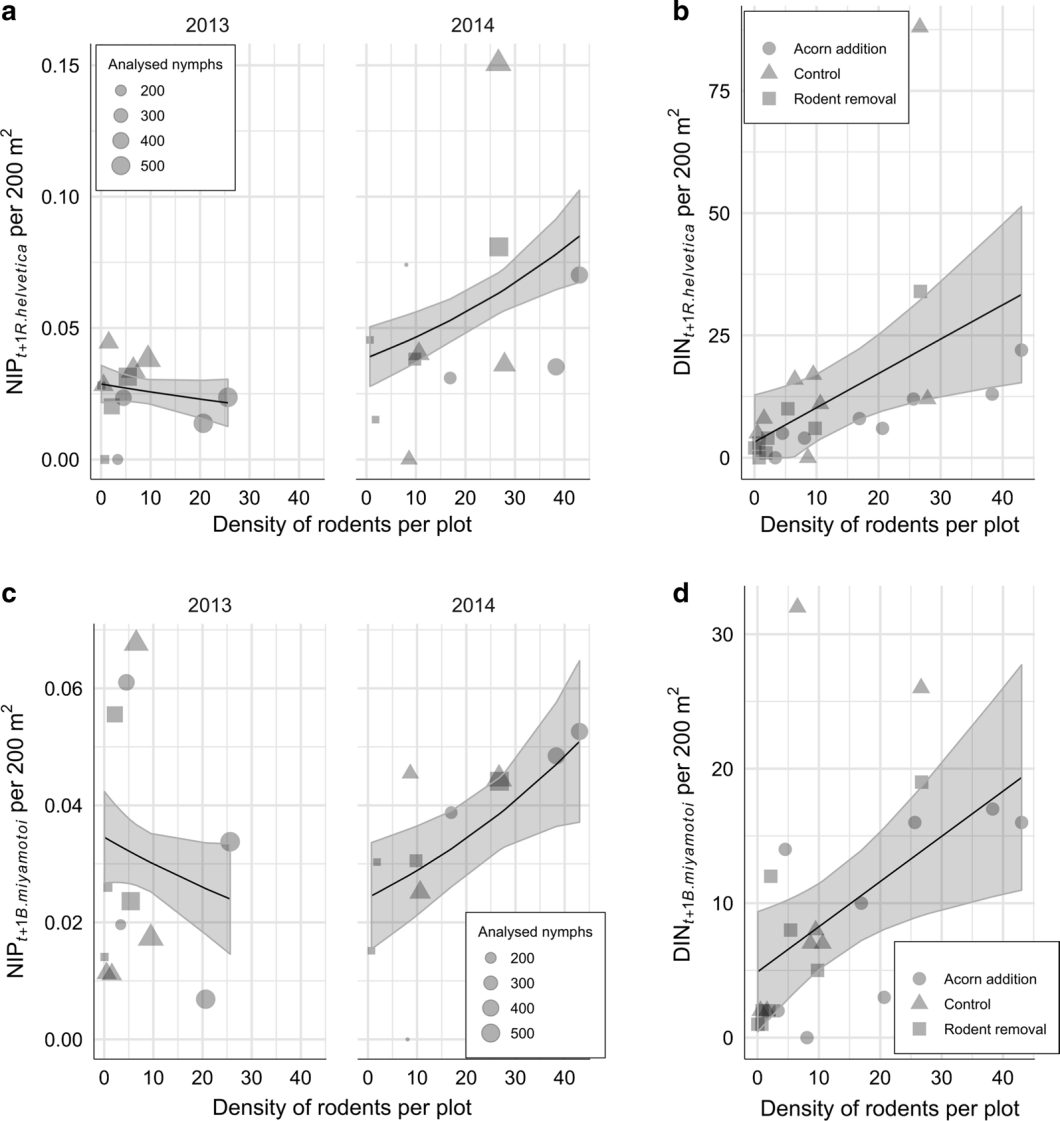
Association between density of rodents and vertically transmitted pathogens. The graphs show the relationship between the number of rodents per plot in year *t* and NIP and DIN (number per 200 m^2^ per plot) in year *t*+1. **a** Effect of rodent density on NIP_*t+*1 *R. helvetica*_. Rodent density had inconsistent effect on NIP (no effect in 2013 and significant positive effect in 2014). **b** Effect of rodent density on DON_*t+*1 *R. helvetica*_. Rodent density had significant positive effect on DIN. **c** Effects of rodent density on NIP_*t+*1 *B. miyamotoi*_. Rodent density had inconsistent effect on NIP (negative but no significant effect in 2013 and significant positive effect in 2014). **d** Effects of rodent density on DON_*t+*1 *B. miyamotoi*_. Rodent density had significant positive effect on DIN

Rodent density had differential effect on NIP_*t+*1 *R. helvetica*_, and a significantly positive effect on DIN_*t+*1 *R. helvetica*_ (*P* = 0.0143; Fig. 9). In case of NIP_*t+*1 *R. helvetica*_, the best-fitting model was a generalized linear model taking into account the differences in association with respect to year (Table 2, Eq. 12). For DIN_*t+*1 *R. helvetica*_, the best model was a simple linear model of rodent density explaining 21% of the variance (Table 2, Eq. 13).

Rodent density had a differential association with NIP_*t+*1 *B. miyamotoi*_ between the years (Fig. 9). In 2013, the association was negative but not significant (*P* = 0.15797) and in 2014, positive and significant (*P* = 0.00862). The association between rodent density and DIN_*t+*1 *B. miyamotoi*_ was significantly positive (*P* = 0.0119; Fig. 9). The best model for NIP_*t+*1 *B. miyamotoi*_ was a generalized linear model of rodent density taking into account the differences in association with respect to year (Table 2, Eq. 6), and for DIN_*t+*1 *B. miyamotoi*_, a simple linear model of rodent density explaining only 22% of the variance (Table 2, Eq. 7).

The association between rodent density and NIP_*t+*1 *S. ixodetis*_ was significantly negative (P < 0.0001) and the best model was a simple generalized linear model of rodent density (Table 2, Eq. 14, Fig. 7). In case of DIN_*t+*1 *S. ixodetis*_, the best model was a linear model of rodent density and (rodent density)^2^, which explained 45% of the variance (Table 2, Eq. 15). The model including a quadratic term allowed revealing significant negative (*P* = 0.005297) non-linear association between rodent density and *S. ixodetis* (Fig. 7).

## Discussion

This study was designed to investigate the association between rodent density and *I. ricinus* nymphs and tick-borne microorganisms. We observed that the densities of rodents affect DON, NIP and DIN in the following year. We found positive associations between rodent density and DON_*t+*1_ regardless of the year and type of treatment (Fig. 5). The NIP_*t+*1_ and DIN_*t+*1_ depending on tick-borne pathogens and microorganisms were associated with the rodent density to a different extent, determined by the infection dynamics of the microorganism species (Figs. 6, 7, 9). In addition, although the treatments affected rodent density in the following years, we did not observe an effect on either the DON (Fig. 2) or DIN_*t+*1 *B. afzelii*_ and DIN_*t+*1 *N. mikurensis*_ (Fig. 3, Additional file 3: Figure S3).

### Rodent density *versus* DON

In all years and plots, we observed a positive association between the DON_*t+*1_ and rodent density, which as a predictor explained 61% of the variance (Fig. 5). Our findings are comparable to previous cross-sectional studies performed in the USA [13, 15] and support that rodents are the main hosts of larval ticks and consistently contribute to a new generation of nymphs in the following year [31]. Regarding the contribution of each rodent species in feeding ticks, wood mice were infested at significantly higher levels with larval ticks than bank voles, which has been reported before [28, 55–58]. The difference in larval tick burden between the two rodent species has been attributed to bank voles acquiring immunity to feeding ticks [59].

### Rodent density *versus* pathogens amplified by rodents

As expected, transmission dynamics of *B. afzelii* and *N. mikurensis* were rodent density-dependent. A higher density of rodents increased the probability for larval ticks to feed on an infected rodent, and subsequently significantly increased the NIP_*t+*1 *B. afzelii*_ and NIP_*t+*1 *N. mikurensis*_ (Fig. 6). Since DON was also rodent density-dependent, there was a strong synergistic effect of rodent density on DIN_*t+*1 *B. afzelii*_ and DIN_*t+*1 *N. mikurensis*_ (Fig. 6). We observed a significantly higher NIP_*N. mikurensis*_ than NIP_*B. afzelii*_. Possibly, *N. mikurensis*-infected rodents are more infectious than while being infected with *B. afzelii*, which may be due to different tissue tropism of these pathogens in the rodent [26, 55]. In addition, *B. afzelii* had a higher infection prevalence in bank voles than in wood mice, which has been reported previously [28, 53, 60–62]. Although one study has reported the opposite, these studies showed that infectivity of voles was much higher than that of mice [28, 55]. As mentioned above, the larval infestation was higher in wood mice whilst a greater proportion of bank voles was infected with *B. afzelii*. This indicates that these two rodent species play distinct but complementary roles in *B. afzelii* transmission dynamics.

There was a significantly negative association between rodent density and NIP_*t+*1 *B. microti*_ and a non-linear association with DIN_*t+*1 *B. microti*_ (Fig. 7). We observed a positive association at low and a negative association at high densities of rodents. We detected the parasite almost exclusively in bank voles; thus, our results might be a consequence of increasing density of wood mouse, which probably is not an amplifying host of *B. microti* (Fig. 4). An alternative explanation for this non-linear association might be that *I. ricinus* is not a main vector of this parasite. Previous studies proposed *I. trianguliceps*, a nidicolous rodent tick species as the main vector [63–65]. It indicates that *B. microti* circulates in the, so called ‘cryptic cycle’ between specialist ticks and rodents, while *I. ricinus* sporadically becomes infected and perhaps acts as an occasional bridge vector to other host species [63].

### Rodent density *versus* a pathogen amplified by birds

An increasing density of rodents negatively associated with NIP_*t+*1 *B. garinii*_ (Fig. 8). This is probably due to the increased number of nymphs uninfected with *B. garinii*, which fed on the widely abundant rodents, *B. garinii-*incompetent hosts [66]. Our plots were not large enough to cover the territory of birds, *B. garinii-*amplifying hosts [35, 67], thus, we speculate that all (or the majority) of the collected *B. garinii*-infected nymphs were brought by birds from outside the experimental plots and that these events were more or less constant during the course of the study. The DIN_*t+*1 *B. garinii*_ remained unaltered which suggests that the increase in the DON eliminated the negative effect of rodents on NIP_*B. garinii*_ (Fig. 8).

### Rodent density *versus* vertically transmitted tick-borne pathogens and microorganisms

We observed a different association between rodent density and the NIP_*t+*1 *R. helvetica*_ depending on the year of study (Fig. 9). Although several studies detected *R. helvetica* in rodent blood and skin samples, and in various ectoparasites feeding on rodents, to date, it is not clear which role rodents play in its transmission cycle [68–70]. Other vertebrates were suggested to be amplifying hosts, for instance, songbirds, which were shown to acquire bacteraemia [35, 36]. Here, we detected *R. helvetica* in rodent ears of both species; however, it is not possible to infer from our results whether rodents acquire systemic infection. On the other hand, we can speculate that *R. helvetica* causes short-term, localized infection in the skin, which is favourable for co-feeding transmission [34], and this transmission route has been attributed to *R. helvetica* on many occasions [35, 71, 72]. The infection prevalence in questing nymphs was significantly higher than in rodents, which indicates that ticks are the main amplification hosts of this bacterium. Rodent density was positively associated with DIN_*t+*1 *R. helvetica*_, which is unexpected and requires further study (Fig. 9).

Rodent density was indifferently associated with NIP_*t+*1 *B. miyamotoi*_ (Fig. 9). In general, the average NIP in questing ticks was only 3% (CI: 2.6–3.4%) and the fluctuations from year to year were small. *Borrelia miyamotoi* is a predominantly vertically transmitted bacterium, which means that a proportion of unfed larvae originating from an infected female tick, may also be infected [73, 74]. The efficiency of the transmission, in an experimental setting, was shown to vary between 6% and 73% [73]. Thus, it is surprising that despite this mode of transmission, the prevalence of *B. miyamotoi* in ticks was not higher. A possible explanation for this could be an inefficient horizontal transmission from infected amplification hosts to naïve ticks as it seems that *B. miyamotoi* does not cause a persistent infection in rodents [75, 76]. Nonetheless, the pathogen can sustain its widespread distribution with only small numbers of ticks being infected. Ultimately, we observed a significant positive effect of rodent density on DIN_*t+*1 *B. miyamotoi*_, possibly related to the general increase in DON (Fig. 9).

An increasing density of rodents was significantly negatively associated with NIP_*t+*1 *S. ixodetis*_ (Fig. 7). Decreasing NIP_*t+*1 *S. ixodetis*_ and increasing DON along with the increasing rodent density resulted in non-linear association between rodents and DIN_*t+*1 *S. ixodetis*_ (Fig. 7). We observed a positive association at low and a negative association at high densities of rodents. Since from the two rodent species investigated in this study we detected *S. ixodetis* almost exclusively in wood mouse, a possible explanation is that increasing bank vole populations diluted the prevalence of this bacterium in ticks (Fig. 4).

Another tick-borne *Spiroplasma* species has been shown to amplify in rodents only in experimental settings [77] and have been reported to cause infections in humans [78, 79]. However, the role of vertebrates in the transmission cycle of S*. ixodetis* in natural conditions is largely unknown. Nevertheless, the detection of *S. ixodetis* in rodent ears indicates that these rodents may facilitate horizontal transfer of the bacterium to naïve ticks. Our findings are in line with a recent phylogenetic study, which has revealed that horizontal transmission is probably one of the drivers responsible for spreading of *S. ixodetis* across tick community [80]. This transmission mode is proposed in addition to the stable vertical transmission, for which spiroplasmas are known [81, 82].

### Effect of treatments on rodent density, DON, and DIN

The variation in rodent density throughout the season was comparable with studies from other woodland areas [83]. The density of both rodent species was affected by food resource availability, here acorns, and with our treatment we succeeded to obtain study sites with significantly different densities of rodents (Fig. 1). It allowed us to study the effect of rodent density on tick population dynamics and associated pathogen infections. Our results with acorn addition are in accordance with previous findings, where bank vole and wood mouse populations increase after mast years [16, 19–22].

Nevertheless, the variation in tick density throughout the years did not follow fluctuations of rodent densities. As a consequence, DON was not affected by our treatment (Fig. 2). There was an effect of the treatment on rodents and an effect of rodents on DON; however, the effect of the treatments on rodents was apparently not enough to establish a significant change in DON. This can also be appreciated from considering the size of the confidence bands in Fig. 5. It is larger than the vertical distance between the treatment lines. In addition, there was no effect of either acorn addition or rodent removal on DIN_*B. afzelii*_ and DIN_*N. mikurensis*_ in the following years (Fig. 3, Additional file 3: Figure S3).

The discrepancy in effect of the treatment indicates that there are additional factors affecting nymphal densities, which expressed high natural variation despite experimental methods. This variation is probably affected by fluctuations in abundance of other vertebrates and/or meteorological conditions affecting seasonal activity of both rodents and ticks. Although in this study we did not assess abundance of other tick hosts, we observed that nymphal activity was affected by temperature, which has been noticed before ([84]; Fig. 2 and Additional file 2: Figure S2). The onset and annual duration of nymphal activity seemed to be related to a number of months with a mean temperature equal or below 7 °C.

### Synchrony in activity of rodents and ticks and its influence on transmission dynamics of tick-borne microorganisms

In our study, rodent density had differential effects on NIP and DIN depending on the species of tick-borne microorganism, which indicates that there are additional factors playing a role in microorganism dynamics. Some of these factors might be timing of both activity and infection of rodents and ticks. In temperate European forests, there is a well-documented synchronization between questing larval ticks and rodents, which facilitates the transition of larvae to nymphs [33, 60, 85]. In addition to driving *I. ricinus* development, rodents contribute to the maintenance of vertically transmitted microorganisms. However, to propagate horizontally transmitted tick-borne pathogens, questing larvae have to be synchronized with infected rodents. Depending on the persistence of a pathogen in a rodent population, rodents may infect larvae directly at the onset of larval activity or after the pathogen has been introduced into the rodent population by infected nymphs. The former situation has been documented for *B. afzelii*, which causes infection in rodents for life, and therefore often persists over winter [60]. In this study, we observed that *B. afzelii*-infected rodents were, indeed, present throughout the year, also before the onset of ticks (Fig. 4).

The latter situation is probably applicable to *N. mikurensis* as the smaller proportion of rodents captured in March was infected with this pathogen, than in later months (Fig. 4). A possible explanation could be that *N. mikurensis* causes systemic blood infection and decreases the overwintering survival of infected rodents. This phenomenon was observed before in bank voles and Puumala virus (PUUV) despite the expectation that hantaviruses have become well adapted to their rodent hosts during co‐evolution [86, 87]. Thus, the most favorable scenario for *N. mikurensis* transmission is synchronization in activity of rodents and infected nymphs right before the onset of larvae [88, 89]. In the Netherlands, nymphs have been shown to start their seasonal activity at least one month before larvae [84], which seems to be advantageous for zoonotic pathogens overwintering in nymphs rather than in vertebrate hosts.

### Study limitations

This study greatly enhanced our understanding about the role of rodents in the dynamics of tick populations and their associated microorganisms. However, we recognize that our semi-experimental approach has logistic limitations on the temporal and spatial extent that must be acknowledged.

First, our results on mechanisms driving the population of nymphs was measured at a relatively small temporal scale, which is only a transition from a larva to a nymph, and do not necessarily hold at a larger scale involving a complete tick life-cycle. A study of many years following all life stages would have added value and perhaps reveal the robustness of a rodent-tick relationship.

Secondly, the size of the plots was not large enough to cover territory of other vertebrate species, such as deer and birds, for which we had no data on density fluctuations. Since these vertebrates may substantially contribute to the tick and pathogen cycles, it is advisable to increase a plot size and obtain data on vertebrate abundance/arrival rate by, for instance, camera trapping [11].

In addition, increasing the plot size would be also beneficial for more accurate description of rodent population dynamics. It has been shown that along with growth and maturation, rodents change their home range, and therefore depending on population structure, they might have various effects on tick and pathogen populations [90].

Furthermore, in the first study year (2012), the experiment of acorn addition was already ongoing, thus we have no good baseline density of rodents to compare the effect of treatments to. It is advisable, in future field experiments, to have a longer monitoring period prior to implementation of the intervention, in order to have a solid baseline in place. This would also increase the statistical power to detect the effects of an intervention.

Lastly, it should be borne in mind that there was natural variation between plots, even within the experimental settings. Hence the data are obtained in a complex environment where rodent densities can vary by plot, year or treatment. Tick population and infection dynamics is intricately interwoven with the rodent dynamics, and we realize that a more involved modelling exercise is probably needed to fully understand the ecology. However, in the current approach our aim was to be ‘descriptiveʼ of responses of ticks and their infection, rather than finding the most appropriate mechanistic model.

## Conclusions

We demonstrated experimentally that increase in rodent density positively affects populations of nymphal ticks in the following year. In addition, we show that prevalence and density of infected ticks with various tick-borne microorganisms are dependent on rodent density to a different extent. These differences probably arouse from varying transmission modes of tick-borne microorganisms and the strongest associations can be observed between rodent density and rodent-associated pathogens that rely on horizontal transmission. Nevertheless, it is not possible to predict disease risk solely on rodent density since we have shown that other factors, independent from our experiment, strongly affected tick density. Our results draw attention to the importance of considering transmission mode of a pathogen as well as other (spatial and temporal) factors while developing models to predict the tick-borne disease risk.

## Supplementary information

**Additional file 1: Figure S1.** An example of an experimental plot 50 × 50 m surrounded by screens to study the effect of rodent density on the density of infected nymphs.

**Additional file 2: Figure S2.** Mean monthly temperature (in °C) from August 2012 to December 2015.

**Additional file 3: Figure S3. a** Density of nymphs infected with *N. mikurensis* (DIN _*N. mikurensis*_) in 2014 and 2015 in all three treatments in comparison to 2013 (baseline year). **b** Differences in DIN _*N. mikurensis*_ between the treatments in two separate years calculated with the Wilcoxon test with a correction for a baseline year (2013). The overall differences between the treatments were not significant either in 2014, or 2015 (*P* = 0.87 and *P* = 0.94, respectively).

**Additional file 4: Table S1.** Daily temperature data from September 2012 to December 2015 collected from the nearest weather station (Deelen, KNMI, the Netherlands). **Text S1.** Primers and probes for detection of *B. microti* and *S. ixodetis*, and qPCR protocol. **Table S2.** Density of nymphs and number of analyzed nymphs for microorganisms per treatment, month and year. **Table S3.** Density of rodents and number of analyzed individuals for microorganisms per rodent species, treatment, month and year. **Table S4.** Prevalences of tick-borne microorganisms in rodents and nymphs. **Table S5.** All tested models for prediction of density of nymphs (DON), nymphal infection prevalence (NIP), and density of infected nymphs (DIN). **Table S6.** Full equations for the best fitting models for prediction of density of nymphs (DON), nymphal infection prevalence (NIP), and density of infected nymphs (DIN).

## Data Availability

Data are available from the corresponding author on request. Unique DNA sequences of *B. burgdorferi* (*s.l*.) were deposited to the GenBank database with the accession numbers MN515318-MN515341.

## Abbreviations

qPCR: quantitative polymerase chain reaction
DNA: deoxyribonucleic acid
KNMI: Koninklijk Nederlands Meteorologisch Instituut
