# Supplementary figures and images for: Effect of rodent density on tick and tick-borne pathogen populations: consequences for infectious disease risk
Source: Parasit Vectors. 2020 Jan 20;13:34. doi: 10.1186/s13071-020-3902-0 (PMC6971888; doi:10.1186/s13071-020-3902-0)

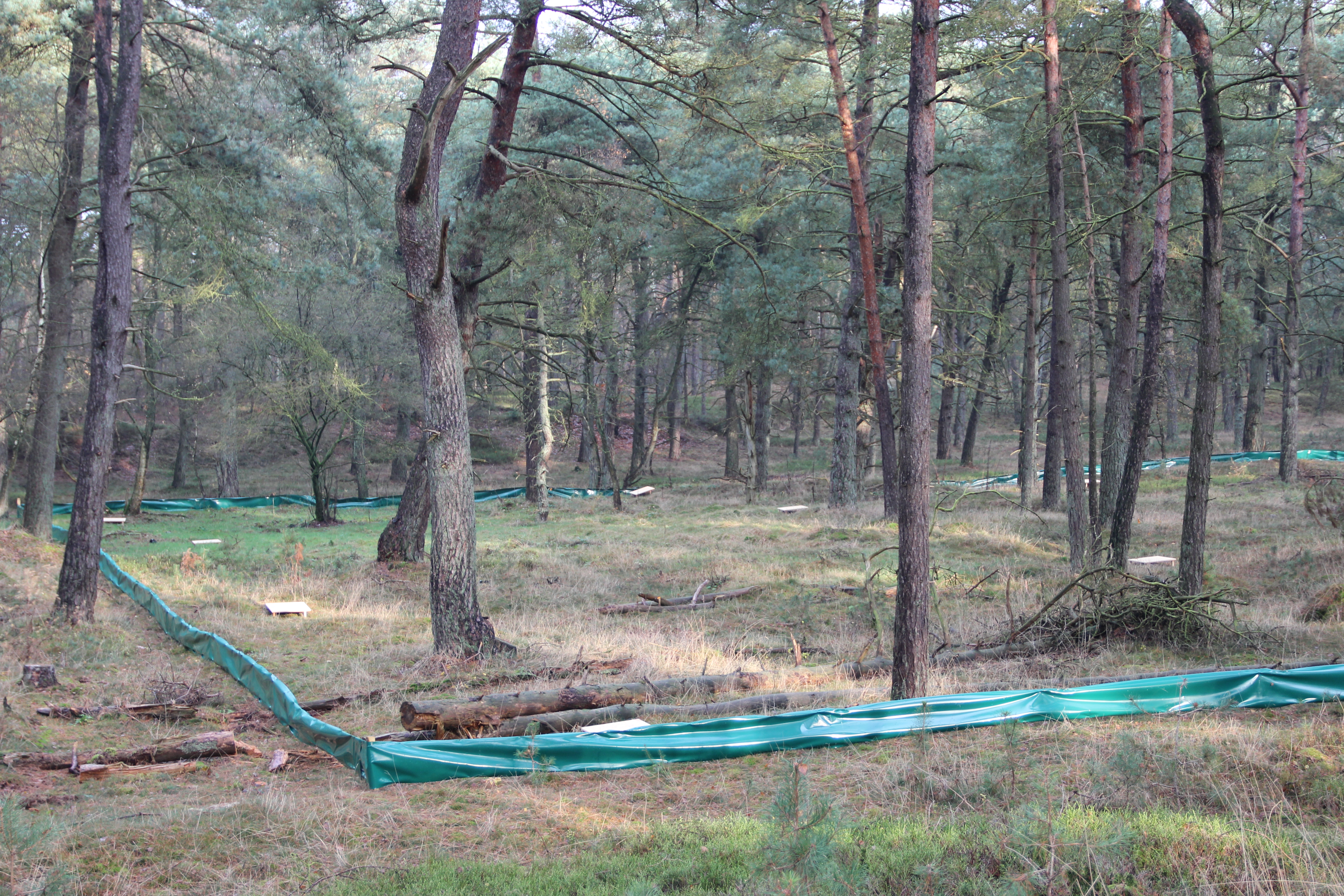

Supplement: Supplementary file 1 — Additional file 1: Figure S1. An example of an experimental plot 50 × 50 m surrounded by screens to study the effect of rodent density on the density of infected nymphs. [file 13071_2020_3902_MOESM1_ESM.jpg]

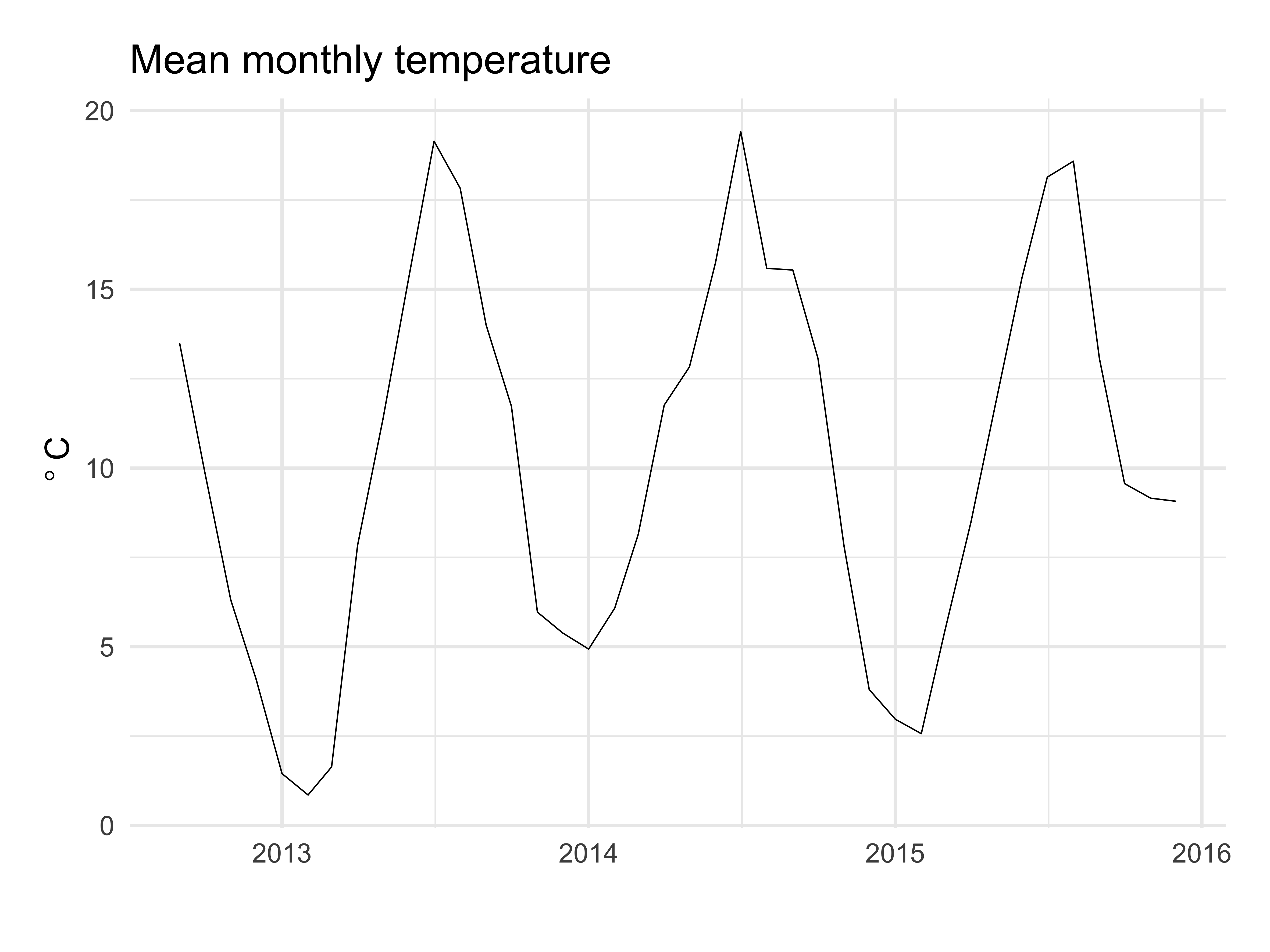

Supplement: Supplementary file 2 — Additional file 2: Figure S2. Mean monthly temperature (in °C) from August 2012 to December 2015. [file 13071_2020_3902_MOESM2_ESM.tiff]

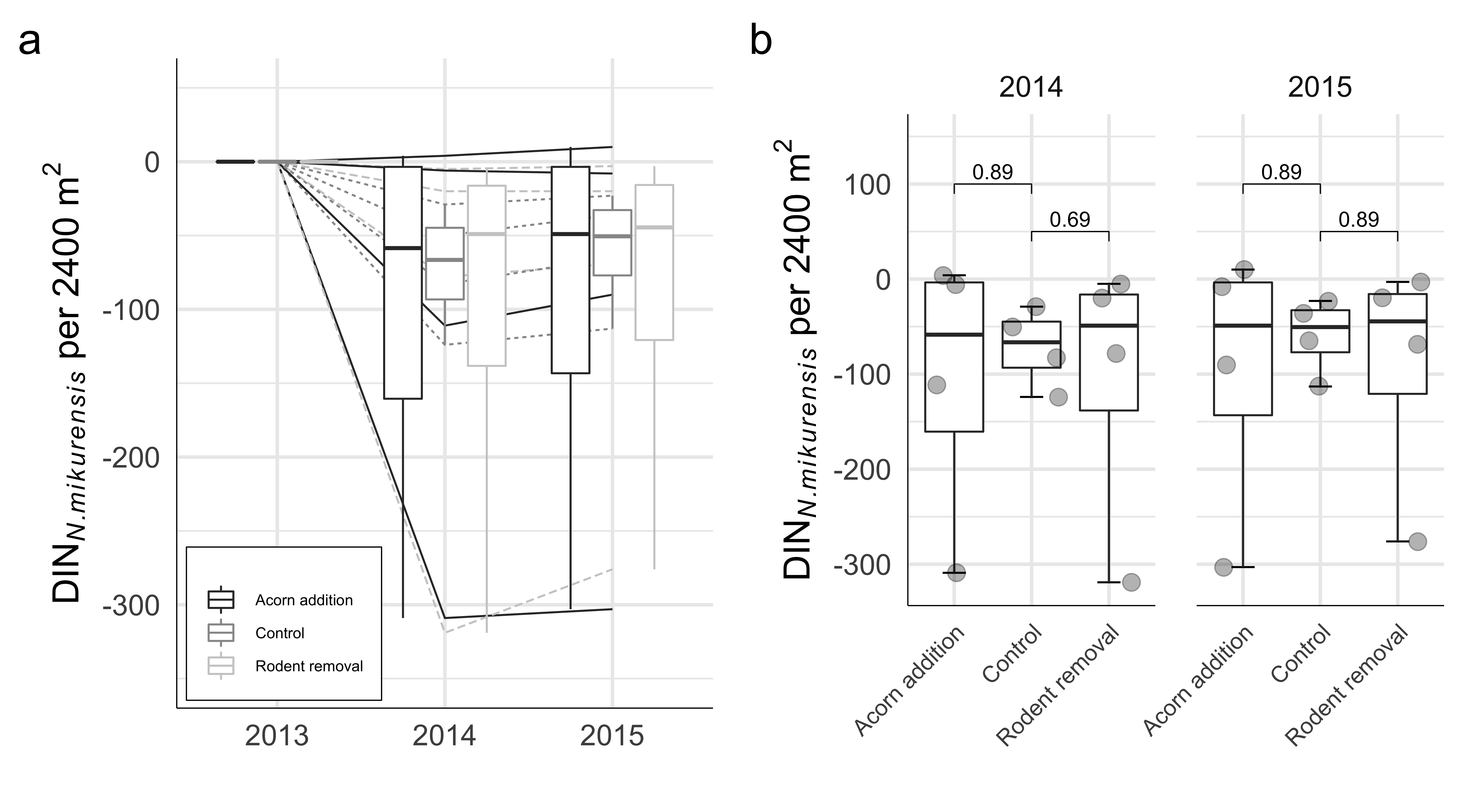

Supplement: Supplementary file 3 — Additional file 3: Figure S3. a Density of nymphs infected with N. mikurensis (DIN N. mikurensis) in 2014 and 2015 in all three treatments in comparison to 2013 (baseline year). b Differences in DIN N. mikurensis between the treatments in two separate years calculated with the Wilcoxon test with a correction for a baseline year (2013). The overall differences between the treatments were not significant either in 2014, or 2015 (P = 0.87 and P = 0.94, respectively). [file 13071_2020_3902_MOESM3_ESM.tiff]
